# Electrocardiographic Repolarization Changes Following Orchiectomy: Insights Into QT Interval Prolongation and Clinical Implications

**DOI:** 10.1111/anec.70217

**Published:** 2026-06-29

**Authors:** Yuta Sakaguchi, Kenichi Iijima, Naomasa Suzuki, Takahiro Hakamata, Yasuhiro Ikami, Sou Otsuki, Nobue Yagihara, Daisuke Izumi, Takayuki Inomata

**Affiliations:** ^1^ Department of Cardiovascular Medicine Niigata University Graduate School of Medical and Dental Sciences Niigata Japan; ^2^ Department of Cardiovascular Biology and Medicine Juntendo University Graduate School of Medicine Tokyo Japan

**Keywords:** orchiectomy, QT interval, sex differences, testosterone

## Abstract

**Introduction:**

Sex steroid hormones influence ventricular repolarization and QT interval duration. Testosterone has been associated with shorter QT intervals, whereas androgen deprivation may prolong repolarization indices. However, the effects of orchiectomy on ECG parameters in humans remain insufficiently characterized.

**Methods:**

We conducted a retrospective analysis of 233 consecutive patients who underwent orchiectomy at our hospital between 2006 and 2021. ECG parameters were compared in 44 patients who had ECG recorded both before and after surgery. Furthermore, the effects of surgery were stratified by unilateral (*n* = 20) and bilateral orchiectomy (*n* = 24) groups to assess differential effects.

**Results:**

The mean age was 59.5 years old. Patients in the unilateral group were significantly younger, taller, and heavier (*p* < 0.05). Orchiectomy was performed for prostate cancer in 22 patients (50%) and for testicular tumors in 19 patients (43%). No significant differences were observed in QRS width before and after surgery. However, QT and corrected QT (QTc) intervals were significantly prolonged following orchiectomy (QT 383 ± 42 to 403 ± 42 ms, *p* < 0.01, QTc 416 ± 35 to 433 ± 35 ms, *p* < 0.001). Post‐surgery, there were no significant differences in the QT and QTc interval prolongation between the two groups.

**Conclusion:**

Orchiectomy was associated with prolongation of QT/QTc intervals, particularly after bilateral orchiectomy. These findings suggest that patients after orchiectomy may be more susceptible to additional factors that prolong ventricular repolarization, including electrolyte abnormalities and QT‐prolonging medications. Careful ECG monitoring may be warranted in selected patients.

## Graphical abstract

QT/QTc interval prolongation was observed following orchiectomy, particularly after bilateral orchiectomy. Careful ECG monitoring may be warranted.

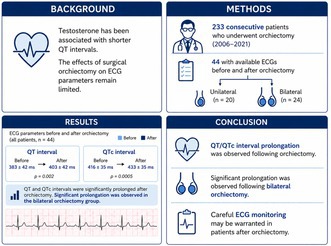

## 1 Introduction

Sex differences in electrocardiographic features and arrhythmia presentation are well recognized (Salama and Bett 2014). Sex steroid hormones modulate cardiac ion channels and influence ventricular repolarization, as reflected by QT interval duration on surface ECG (Itoh et al. 2016). Testosterone has been associated with shorter QT intervals, whereas androgen deprivation may prolong ventricular repolarization indices (Bidoggia et al. 2000). Medical or surgical castration is commonly used to manage androgen‐sensitive conditions, and androgen deprivation therapy for prostate cancer has been associated with QT interval prolongation and ventricular arrhythmias (Hasegawa et al. 2021). Excessive QT prolongation may predispose patients to life‐threatening ventricular arrhythmias, including torsades de pointes and ventricular fibrillation (Shimizu and Horie 2011; Shimizu 2013; Hayashi et al. 2015). Because serum androgen levels markedly decrease after orchiectomy, similar ECG changes may occur after surgical castration (Oefelein et al. 2000; Wiechno et al. 2017). However, data specifically evaluating ECG changes after surgical orchiectomy remain limited. Therefore, this study aimed to investigate changes in ECG parameters before and after orchiectomy, with a particular focus on QT/QTc intervals.

## 2 Methods

### 2.1 Study Population

This retrospective observational study included 223 consecutive patients who underwent unilateral or bilateral orchiectomy at Niigata University Medical and Dental Hospital between January 2006 and December 2021. Of these, 44 patients with available 12‐lead ECG recordings both before and after orchiectomy were included in the analysis. Patients receiving medications known to affect QT interval duration were excluded to minimize confounding effects on ventricular repolarization assessment. The median interval between orchiectomy and follow‐up ECG was 631 days (interquartile range, 96–1373 days).

### 2.2 Data Analysis

QT intervals were primarily measured in lead II using the tangent method. When the end of the T wave could not be clearly identified in lead II, leads V5 or V6 were used. Measurements were performed using a semi‐automated digitizing program with electronic calipers by an experienced observer who was blinded to all clinical information. The corrected QT interval (QTc) was calculated using Bazett's formula (Bazett 1920).

### 2.3 Statistical Analysis

Continuous variables are expressed as the mean ± standard deviation (SD), while categorical variables are presented as numbers and percentages. Comparisons between groups were conducted using the χ^2^ test for categorical variables and the Student *t*‐test for continuous variables. Differences in the parameters before and after orchiectomy were analyzed using a paired *t*‐test. The Pearson correlation coefficient was used to assess the relationship between QTc interval and other variables. All statistical analyses were performed using SPSS version 25 (IBM Japan, Tokyo, Japan). Statistical significance was set at *p* < 0.05.

### 2.4 Consent

Informed consent was obtained using an opt‐out approach. Information regarding the study was disclosed on the institutional website, and participants were provided with the opportunity to decline participation.

## 3 Results

### 3.1 Baseline Characteristics

Table 1 presents the baseline characteristics of the 44 patients stratified into the unilateral and bilateral orchiectomy groups. The mean age of the patients was 59.5 years, and all patients were male. Testicular cancer was the most common indication for orchiectomy in the unilateral group (*n* = 18, 90%) and prostate cancer was the most common indication in the bilateral group (*n* = 22, 92%). No patients had documented arrhythmias, including polymorphic ventricular tachycardia.

**TABLE 1:** Patient characteristics.

Variable	All (*n* = 44)	Unilateral (*n* = 20)	Bilateral (*n* = 24)	*p*
Age (years)	59.5 ± 15.8	47.0 ± 15.0	69.9 ± 6.54	< 0.0001
Male sex, *n* (%)	44 (100)	20 (100)	24 (100)	1
Body Height (cm)	167 ± 7.4	170 ± 5.8	162 ± 6.7	0.00018
Body Weight (kg)	61.4 ± 10.1	65.5 ± 11.2	58.6 ± 8.1	0.02
Systolic blood pressure (mm Hg)	126.3 ± 18.6	131 ± 19	126 ± 19	0.34
Diastolic blood pressure (mm Hg)	75.8 ± 15.1	81.4 ± 16.5	72.1 ± 12.7	0.02
Comorbidities				
Hypertension, *n* (%)	18 (40)	5 (25)	13 (54)	0.10
Diabetes mellitus, *n* (%)	7 (16)	1 (5)	6 (25)	0.16
Angina pectoris, *n* (%)	1 (2)	0 (0)	1 (4)	1
Atrial fibrillation, *n* (%)	4 (9)	3 (15)	1 (4)	0.47
Electrolyte				
Sodium (mEq/L)	139 ± 2.5	139 ± 2.1	140 ± 2.8	0.34
Potassium (mEq/L)	4.1 ± 0.5	3.9 ± 0.4	4.2 ± 0.4	0.01
Chloride (mEq/L)	104 ± 3.1	103 ± 2.9	103 ± 2.2	0.31
Magnesium (mEq/L)	1.75 ± 0.13	1.73 ± 0.11	1.72 ± 0.14	0.95
Calcium (mg/dL)	9.34 ± 0.44	9.39 ± 0.54	9.21 ± 0.31	0.22
Medication				
Beta‐adrenergic blocker, *n* (%)	4 (9)	0 (0)	4 (17)	0.11
ACE‐I/ARB, *n* (%)	7 (16)	1 (5)	6 (25)	0.11
Calcium channel blocker, *n* (%)	7 (16)	1 (5)	6 (25)	0.11
Etiology				
Prostate cancer, *n* (%)	22 (50)	0 (0)	22 (92)	< 0.0001
Testicular tumor, *n* (%)	20 (45)	19 (95)	1 (4)	< 0.0001
ECG findings at baseline				
Heart Rate, (beats/min)	72 ± 16	74 ± 17	71 ± 16	0.56
PR interval, (ms)	161 ± 27	158 ± 27	163 ± 27	0.52
QRS duration, (ms)	106 ± 15	105 ± 10	107 ± 18	0.52
QT interval, (ms)	383 ± 42	378 ± 39	387 ± 45	0.50
Corrected QT interval, (ms)	416 ± 35	412 ± 34	418 ± 36	0.54

Abbreviations: ARB, angiotensin II receptor blocker; ACE‐I, angiotensin converting enzyme inhibitor.

There were no significant differences in the plasma levels of sodium, chloride, calcium, and magnesium between the two groups. However, the plasma potassium level was significantly lower in the unilateral group (*p* = 0.01), though it remained within the normal range.

At baseline, the QT and QTc intervals were 383 ± 42 ms and 416 ± 35 ms, respectively, with no significant difference between the unilateral and bilateral groups.

### 3.2 Change in the Electrocardiographic Parameters

In all patients, the heart rate and QRS duration were similar at baseline and after orchiectomy (Table 2). However, both the QT and QTc intervals were significantly prolonged after orchiectomy compared to baseline (QT interval: 383 ± 42 ms to 403 ± 42 ms, *p* = 0.002, QTc interval: 416 ± 35 ms to 433 ± 35 ms, *p* = 0.0005).

**TABLE 2:** Change in the ECG parameters in all patients.

Parameters	Before orchiectomy	After orchiectomy	*p*
Herat Rate, per mm	72 ± 16	72 ± 15	0.97
PR interval, ms	161 ± 27	166 ± 31	0.048
QRS duration, ms	106 ± 15	105 ± 16	0.38
QT interval, ms	383 ± 42	403 ± 42	0.002
QTc interval, ms	416 ± 35	433 ± 35	0.0005

Abbreviation: QTc, corrected QT.

### 3.3 Comparison of Changes in ECG Parameters Between Unilateral and Bilateral Groups

**FIGURE 1:**
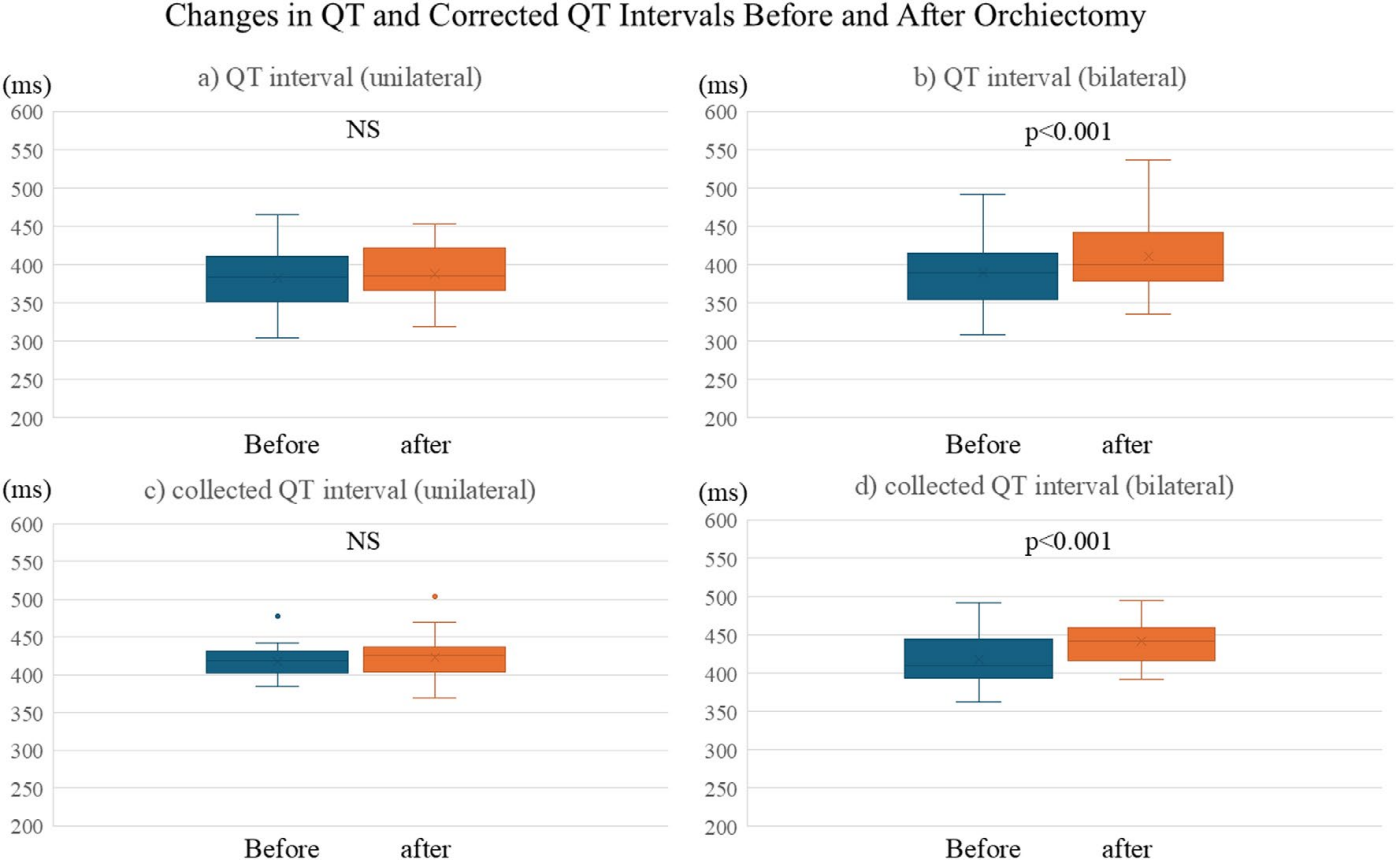
Changes in QT and Corrected QT Intervals Before and After Orchiectomy. QT intervals (a and b) and corrected QT (QTc) intervals (c and d) measured before and after orchiectomy are shown separately for the unilateral and bilateral orchiectomy groups. No significant changes in QT or QTc intervals were observed after unilateral orchiectomy. In contrast, significant prolongation of both QT and QTc intervals was observed following bilateral orchiectomy.

We further investigated the differences in changes to ECG parameters between the unilateral and bilateral groups (Table 3). In the unilateral group, the PR interval remained stable, showing no significant change between 158 ± 27 ms and 159 ± 28 ms (*p* = 0.38). In contrast, the PR interval in the bilateral group significantly prolonged from 163 ± 27 ms to 172 ± 34 ms (*p* = 0.02). For the unilateral group, the QT and QTc intervals showed a trend toward prolongation, with values ranging from 382 ± 41 ms to 392 ± 30 ms (*p* = 0.08) and 412 ± 34 ms to 424 ± 31 ms (*p* = 0.13), respectively, though these changes were not significantly different. Conversely, in the bilateral group, both the QT and QTc were significantly prolonged from 387 ± 45 ms to 412 ± 49 ms (*p* < 0.001) and 418 ± 36 ms to 441 ± 37 ms (p < 0.001), respectively (Figure 1).

**TABLE 3:** Comparison with change in the ECG parameters between unilateral and bilateral.

Parameters	Unilateral group (*n* = 20)			Bilateral group (*n* = 24)		
	Pre	Post	*p*	Pre	Post	*p*
Herat Rate, per mm	74 ± 17	73 ± 16	0.85	71 ± 16	72 ± 15	0.79
PR interval, ms	158 ± 27	159 ± 28	0.38	163 ± 27	172 ± 34	0.02
QRS duration, ms	105 ± 10	104 ± 8.6	0.31	107 ± 18	106 ± 20	0.77
QT interval, ms	378 ± 41	392 ± 30	0.08	387 ± 45	412 ± 49	< 0.001
Corrected QT interval, ms	412 ± 34	424 ± 31	0.13	418 ± 36	440 ± 37	< 0.001

## 4 Discussion

### 4.1 Main Findings

This study analyzed 44 orchiectomy cases and reported the following findings: (1) orchiectomy prolonged the QTc interval in 68% of patients; (2) the degree of QT prolongation was more pronounced in the bilateral orchiectomy group, and (3) orchiectomy did not affect sinus node function or heart depolarization as reflected by heart rate and QRS duration.

### 4.2 Effects of Orchiectomy on the Cardiac Depolarization

Although the PR interval was significantly prolonged in the bilateral group, the absolute value of this prolongation was limited to less than 10 ms. Basic studies have not been conducted on this mechanism, and its cause remains unclear. Notably, the bilateral group included older patients, suggesting that aging may be involved in this change. However, further experimental and clinical research is needed to clarify this phenomenon.

### 4.3 Effects of Orchiectomy on the Cardiac Repolarization

In this study, the QT and QTc intervals were significantly prolonged after orchiectomy. Testosterone has been reported to increase I_Ks_ (Bai et al. 2005) and decrease I_Ca_ (Masuda et al. 2018) shorten APD, and subsequently reduce the QT interval. Basic studies (Salem et al. 2019) suggest that androgen deprivation enhances the late sodium channel current, contributing to APD prolongation and may be involved in QT prolongation in clinical cases. The bilateral group had a more pronounced effect on QT prolongation compared to the unilateral group. Unilateral orchiectomy is often performed for testicular tumors, while bilateral orchiectomy is used for surgical castration to manage prostate cancer. A serum testosterone value of 0.5 ng/mL or less was used to define “castrate level” based on serum assay methods developed in the 1960s (Burger et al. 1964). Following bilateral orchiectomy, serum testosterone levels were found to decrease to 0.15 ng/mL, whereas unilateral orchiectomy resulted in levels of approximately 3.6 ng/mL (Oefelein et al. 2000; Wiechno et al. 2017). Although testosterone levels were not measured in the present study, the greater reduction in testosterone after bilateral orchiectomy may partly explain the more pronounced QT interval prolongation observed in this group.

### 4.4 Clinical Perspectives

Although no cases of sudden deaths due to fatal arrhythmias were observed in this study, QT interval prolongation can trigger ventricular arrhythmias including polymorphic ventricular tachycardia and ventricular fibrillation (Hayashi et al. 2015). In prior studies, fatal arrhythmias were reported in 1.3% of patients due to QT prolongation induced by medical castration for prostate cancer treatment (Hasegawa et al. 2021). The findings of this study suggest that careful ECG monitoring of the QT interval is recommended in post‐orchiectomy patients to prevent the risk of sudden cardiac death due to fatal arrhythmias. This is especially important in situations that may further prolong the QT interval, such as the use of drugs that cause QT prolongation, electrolyte abnormalities (hypokinemia, hypomagnesemia, etc.), and bradycardia.

### 4.5 Study Limitation

This study has several limitations. First, although the study population was derived from a consecutive orchiectomy cohort, only patients with available ECG recordings both before and after orchiectomy were eligible for inclusion. Therefore, selection bias cannot be excluded and the generalizability of the present findings may be limited. Second, this was a single‐center retrospective study with a relatively small sample size. Third, the timing of post‐orchiectomy ECG acquisition varied across patients, with a median interval of 631 days (interquartile range, 96–1373 days). Because of the limited sample size, subgroup analyses according to ECG timing could not be performed. Fourth, plasma testosterone levels were not measured, limiting the ability to directly correlate testosterone reduction with QT prolongation. Finally, interpretation of differences between unilateral and bilateral orchiectomy should be made with caution. The two groups differed with respect to age and underlying disease, with testicular cancer predominating in the unilateral group and prostate cancer predominating in the bilateral group. In addition, potential differences in comorbidities and concomitant therapies may have influenced the observed ECG findings. Therefore, the observed differences cannot be attributed solely to the extent of orchiectomy.

In addition, robust multivariable analysis adjusting for age, indication for orchiectomy, electrolyte levels, timing of ECG acquisition, comorbidities, and concomitant therapies could not be performed because of the limited sample size.

## 5 Conclusions

Orchiectomy was associated with prolongation of QT/QTc intervals. Significant QT/QTc interval prolongation was observed following bilateral orchiectomy. These findings suggest that patients after orchiectomy may be more susceptible to additional factors that prolong ventricular repolarization, including electrolyte abnormalities and QT‐prolonging medications. Therefore, careful monitoring of the QT interval may be warranted in selected patients following orchiectomy.

## Author Contributions

Yuta Sakaguchi: conceptualization, data collection, statistical analysis, interpretation of data, and drafting of the manuscript. All co‐authors contributed to data interpretation and critical revision of the manuscript. All authors read and approved the final manuscript.

## Funding

The authors have nothing to report.

## Conflicts of Interest

The authors declare no conflicts of interest.

## Data Availability

The data that support the findings of this study are available from the corresponding author upon reasonable request.
